# Stress Hormone Corticosterone Controls Metabolic Mitochondrial Performance and Inflammatory Signaling of In Vitro Cultured Sertoli Cells

**DOI:** 10.3390/biomedicines10092331

**Published:** 2022-09-19

**Authors:** Ana M. Silva, Carina T. Ribeiro, Raquel L. Bernardino, Ivana Jarak, Rui A. Carvalho, M. A. Pereira-Sampaio, Diogo B. de Souza, Marco G. Alves, Pedro F. Oliveira

**Affiliations:** 1 Department of Anatomy and Unit for Multidisciplinary Research in Biomedicine (UMIB), School of Medicine and Biomedical Sciences (ICBAS), University of Porto, 4050-313 Porto, Portugal; 2 Urogenital Research Unit, Rio De Janeiro State University, Rio De Janeiro 20551-030, Brazil; 3 Department of Pharmaceutical Technology, Faculty of Pharmacy, Azinhaga de Santa Comba, Pólo III–Pólo das Ciências da Saúde, University of Coimbra, 3000-548 Coimbra, Portugal; 4 Department of Life Sciences, Faculty of Sciences and Technology, University of Coimbra, 3030-790 Coimbra, Portugal; 5 LAQV-REQUIMTE and Department of Chemistry, University of Aveiro, 3810-193 Aveiro, Portugal

**Keywords:** male fertility, Sertoli cell, stress, corticosterone, metabolism, mitochondria, interleukine-6

## Abstract

Stress, as a physiological response, is a major factor that affects several processes, including reproductive functions. The main hormonal players of stress are cortisol (humans) and corticosterone (rodents). Sertoli cells (SCs), as key contributors for the testicular homeostasis maintenance, are extensively challenged by different hormones, with glucocorticoid corticosterone being the signaling modulator that may impact these cells at different levels. We aimed to characterize how corticosterone modulates SCs energy balance, putting the mitochondrial performance and signaling output in perspective as the cells can disperse to the surroundings. TM4 mouse SCs were cultured in the absence and presence of corticosterone (in nM: 20, 200, and 2000). Cells were assessed for extracellular metabolic fluxes, mitochondrial performance (cell respirometry, mitochondrial potential, and mitochondrial complex expressions and activities), and the expression of androgen and corticosteroid receptors, as well as interleukine-6 (IL-6) and glutathione content. Corticosterone presented a biphasic impact on the extracellular fluxes of metabolites. Low sub-physiological corticosterone stimulated the glycolytic activity of SCs. Still, no alterations were perceived for lactate and alanine production. However, the lactate/alanine ratio was decreased in a dose-dependent mode, opposite to the mitochondrial complex II activity rise and concurrent with the decrease of IL-6 expression levels. Our results suggest that corticosterone finely tuned the energetic profile of mouse SCs, with sub-physiological concentrations promoting glycolytic expenditure, without translating into cell redox power and mitochondrial respiratory chain performance. Corticosterone deeply impacted the expression of the pro-inflammatory IL-6, which may alter cell-to-cell communication in the testis, in the last instance and impact of the spermatogenic performance.

## 1. Introduction

Corticosterone is the main glucocorticoid in most mammals, with it being described as a whole-body energy balancer. It regulates several metabolic pathways, as well as immune cascades and triggering responses, that are attributed to the physiological manifestation of stress [1]. Indeed, glucocorticoids regulate the availability of energy via modulation of gluconeogenesis, glucose expenditure, fat, and protein metabolism [2,3]. Glucocorticoid secretion is a classic endocrine response to stress. Increased glucocorticoid production induces gluconeogenesis to maintain the circulating glucose levels necessary to respond to stress. When the production of glucocorticoids is increased, an imbalance occurs in various organs, as well as the reproductive organs. Glucocorticoids affect testicular function at several levels: (1) in the hypothalamus, they decrease the synthesis and release of GnRH; (2) in the pituitary, which inhibits the synthesis and release of luteinizing hormone (LH) and follicle-stimulating hormone (FSH); (3) in the testis, which directly modulates steroidogenesis and gametogenesis [4]. Although it has been described that normal male reproductive function requires residual levels of glucocorticoids and the activation of the associated receptors [1], it is documented that corticosteroids negatively impact males’ fertility, with a particular focus on the Sertoli cells (SCs). SCs are the somatic cells that are responsible for the compartmentalization of the testis and support of spermatogenesis. Adjacent SCs are connected, creating a blood–testis barrier (BTB), which allows them to create a protected environment within the seminiferous tubules. These somatic cells play five essential roles that allow for the occurrence of the spermatogenic event: (1) creation of the BTB; (2) nutritional and structural support to the developing germ cells; (3) elimination of non-viable germ cells; (4) production of fluid tubular seminiferous; (5) creation of an immune-privileged environment [5].

In general, glucocorticoid receptors activity regulates testicular function, thus modulating the circulating levels of FSH and LH and impacting the ratio between the SCs and germ cells [6]. At the physiological level, corticosterone effects on mouse SCs have never been described, especially considering their impact on metabolic fitness, in general, and in mitochondria, in particular, as well as the effects that could alter the way these cells communicate with neighboring cells. The commitment of energetic management and oxidative stress underlying the mitochondrial activity, particularly in respiratory chain electron flux and ATP synthesis coupling, is a major question to be addressed upon corticosterone treatment. SCs present a crucial metabolic role, based on aerobic glycolysis, to produce lactate to feed developing germ cells [7]. Still, mitochondria do not become totally dysfunctional, with their activity being maintained to restore intermediate metabolites and the pools of ATP for the SC’s use, as well as to balance its redox power [8].

The deviation from baseline corticosterone values and possible alteration in the control of the release of certain cytokines [9] can also impact the normal performance of testicular homeostatic management, which is attributed, in large part, to SCs. Minimal levels of interleukin-6 (IL-6) are needed to trigger the essential actions (autocrine and paracrine) in testicular physiology and maintain healthy male fertility. However, in some way, if these interleukin levels deviate from the predicted physiological levels, it may impact the full process, regarding spermatogonia maintenance and maturation [9]. IL-6 was reported to inhibit the synthesis of DNA in a specific phase of meiosis in spermatocytes, maybe working as a paracrine regulator of the seminiferous epithelium and negatively controlling germ cell DNA replication [10]. Considering the major relevance of SCs in the structural support, nurturing, and innate protection against agents external to the testicular environment of spermatogonia, the local production of pro-inflammatory cytokines in the testis (by SCs) can be of major importance. IL-6 is also described to regulate the BTB by perturbing the integrity of the BTB and altering the normal localization and steady-state levels of the BTB integral membrane proteins [11]. In other species and tissues, interesting correlations between the metabolic performance and redox state were reported with inflammatory signaling, including IL-6 [12].

Considering the multiple targets for corticosterone action described above, we considered a panel of corticosterone concentrations, in order to expose the mouse SCs, based on the literature [13,14,15], and evaluated their glycolytic and mitochondrial performance, as well as their autoimmune activity, through the expression of IL-6.

## 2. Materials and Methods

### 2.1. Chemicals

Fetal bovine serum (FBS) was obtained from Merck-Millipore (Berlin, Germany). Insulin–transferrin–sodium selenite (ITS) supplement was purchased from Life Technolgies (Gaithersburg, MD, USA). Pierce BCA protein assay kit was obtained from Thermo Scientific (Waltham, MA, USA). Clarity™ western ECL substrate was purchased from Bio-Rad (Bio-Rad, CA, USA). NZYColour Protein Marker II, NZY M-MuLV reverse transcriptase (M-MLV RT), random hexamer primers, dNTPs, NZYTaq green master mix, Greensafe and NZY qPCR green master mix, and NZYDNA Ladder VI were obtained from NZYTech (Lisbon, Portugal). Corticosterone (with 98.5% purity minimum) and all other chemicals were purchased from Sigma–Aldrich (St. Louis, MO, USA). Other specific reagents are described alongside their respective methods.

### 2.2. Sertoli Cell Line

Assays were performed using immortalized SCs from mouse (TM4) (CRL-1715™), previously purchased from ATCC (LGC Standards, Middlesex, UK). Cells were cultured and handled accordingly with ATTC protocol and original guidelines [16]. SCs culture medium was a 1:1 mixture of DMEM:Ham’s F12, supplemented with 10% heat-inactivated FBS, 25 mg/mL gentamicin, 100 U/mL penicillin, 100 µg/mL streptomycin sulfate, 2.5 µg/mL amphotericin B, 15 mM HEPES, and sodium bicarbonate, with pH adjusted to 7.4. Cells were plated on standard surface treated plates and incubated at 37 °C, 5% CO_2_.

### 2.3. Experimental Groups and Sample Collection

TM4 SCs were cultivated as described above, until reaching 70–80% confluence before corticosterone treatment. Then, cells were washed thoroughly with PBS, and the culture medium was replaced by serum-free DMEM:F12 (1:1) medium, with ITS supplementation (insulin 10 mg/L; transferrin 5.5 mg/L; sodium selenite 6.7 μg/L, pH 7.4), and supplemented with three different corticosterone concentrations (in nM: 20, 200, and 2000). The chosen concentrations were based on those described in mice serum (sub, iso, and supra-physiological concentrations, respectively) [13,14,15]. Additionally, we considered a condition without corticosterone (CORT-free) to perform data normalization. After 24-h exposure, cells were used to perform, i.e., 1H-NMR metabolic profiling (collecting and freezing 0.5 mL of culture medium), protein and RNA extraction by cellular pellet, intact cell respirometry [17], and mitochondria isolation [18], in order to assess complex I and II activities [19,20]. Additional TM4 cell cultures in 6- and 96-well plates, under the same culture conditions, were used to address glutathione content [21] and mitochondrial membrane potential [17], respectively. The TM4 cell line used to make these experimental groups was between passages 10 and 20.

### 2.4. Evaluation of Cellular Metabolic Performance by Proton Nuclear Magnetic Resonance Spectroscopy

TM4 SCs were cultivated as described previously, considering 24 h treatment with corticosterone. At time zero of incubation 0.5 mL of medium was collected (corresponding to basal composition, with no metabolization) and freeze-clamped in liquid nitrogen for later 1H-NMR analysis. After 24 h treatment, 0.5 mL of medium from each plate were collected and freeze clamped. The 1H-NMR spectra were acquired at 14.1 Tesla, at 25 °C, using a Varian (Varian Inc, Palo Alto, CA, USA) 600 MHz spectrometer, equipped with a 3 mm indirect detection probe, to determine metabolite variation in the different experimental groups during the time-course of the experiment using previously described methods [7], with minor modifications. In brief, each 1H-NMR spectra consisted of 21.5 k points, defining a 7.2 kHz spectral width. A minimum of 64 scans were averaged using an interpulse delay of 10 s, as well as a 30° radiofrequency pulse, to ensure full relaxation of magnetization towards quantitative analysis. Sodium fumarate was used as an internal reference (singlet, 6.50 ppm) to quantify the metabolites in the solution: lactate (doublet, 1.33 ppm), alanine (doublet, 1.45 ppm), succinate (singlet, 2.393 ppm), glutamine (triplet, 3.766 ppm), and H1-α glucose (doublet, 5.22 ppm). The relative areas of 1H-NMR resonance were quantified using the curve-fitting routine supplied with the NUTSpro NMR spectral analysis program (Acorn, NMR Inc., Fremont, CA, USA). Before Fourier transform, each FID was zero-filled and multiplied by a 0.2 Hz Lorentzian. Metabolite consumption (or production) per cell was calculated by measuring the accumulated variation of the metabolite (versus time zero) after 24 h and dividing it by the total number of cells in each plate [7].

### 2.5. Oxygen Consumption Rate Measurements in Intact Cells

Cells were cultivated until reaching around 70–85% confluence. Prior to oxygen measurements, cells were trypsinized, resuspended in respiration medium (specific for SCs), and counted in a Neubauer chamber to address cell density. Respiration medium was based on cell culture medium, with a total of 17.5 mM glucose and 4 mM L-glutamine, without serum, and supplemented with 1% ITS (0.01 mg/mL recombinant human insulin, 0.0055 mg/mL human transferrin, substantially iron-free, and 0.005 μg/mL sodium selenite). To assess the oxygen consumption rates we performed open-air oxygen measurements in a thermostatized (by a water jacket, at 37 °C) 1 mL volume chamber, using a Clark-type electrode from Hansatech Oxytherm System, connected to Oxytrace Plus data acquisition software. Respirometry assay was adapted from different protocols [18,22]. Each assay started with a 30 min initial respiration, until it reaches a steady-state, with oxygen consumption rate (OCR) measured in the last 5 min. Then, the sequential addition of mitochondrial modulators, such as 2 µg/mL oligomycin, 2 µM FCCP (Carbonyl cyanide-p-trifluoromethoxyphenylhydrazone) and a mixture of 0.5 µM rotenone + 2.5 µM antimycin A, was performed (each modulation period lasting, on average, 15 min) to assess the rates needed to calculate the parameters: basal respiration (Initial respiration minus Rotenone+Antimycin A state), maximal respiration (FCCP state minus Rotenone+Antimycin A state), ATP turnover (Initial respiration minus Oligomycin state), proton leak (Oligomycin state minus Rotenone+Antimycin A state), spare respiratory capacity (FCCP state minus Initial respiration), and non-mitochondrial respiration (Rote-none+Antimycin A state). All values were normalized to 1 × 10^6^ cells. The parameter analysis was adapted to the polarographic measurement of oxygen from the literature [22,23].

### 2.6. Mitochondrial Membrane Potential (ΔΨm) Assay in Intact Cells

The measurement of mitochondrial membrane potential (ΔΨm) was performed by fluorescence detection of a cationic dye, JC-1 (1,1′,3,3′-Tetraethyl-5,5′,6,6′-tetrachloroimidacarbocyanine iodide), that migrates to negatively polarized mitochondria, using a protocol adapted from [24,25]. Briefly, cells were seeded in 96-well black side clear bottom microplates and treated with different concentrations of corticosterone for 24 h. At the end of treatment, cells were incubated with JC-1 (3 μL/10 mL) for 30 min at 37 °C, 5% CO_2_. Afterwards, cells were washed with Hank’s balanced salt solution (HBSS) supplemented with 17.5 mM glucose and 10 mM HEPES, as well as with calcium and magnesium salts. Fluorescence was determined (two-wavelength pairs: Ex485/Em528 nm and Ex530/Em590 nm, JC-1 monomers and aggregates, respectively) using a Synergy™ HTX multi-mode microplate reader, Bi-oTek (Winooski, VT, USA). Fluorescence ratio (JC-1 aggregates/monomers) was calculated to assess changes in ΔΨm, which can represent mitochondrial depolarization or hyperpolarization. Data was normalized to values obtained from non-treated cells.

### 2.7. Isolation of Mitochondria

TM4 SCs were treated over 24 h with corticosterone, as described above. Cells were trypsinized, and freshly obtained pellets were homogenized with Glass/Teflon Potter–Elvehjem (approximately 20 strokes) in 2 mL ice cold buffer (130 mM sucrose, 50 mM KCl, 5 mM MgCl_2_, 5 mM KH_2_PO_4_, 5 mM HEPES; pH 7.4, and 0.01% digitonin) to promote the rupture of cells. Mitochondria-rich fractions were obtained after differential centrifugations, at 4 °C (800× *g*, 10 min, pellet down cell debris, recovering supernatant; 12,000× *g*, 15 min, recovering pellet, washed with ice cold buffer—250 mM sucrose, 5 mM HEPES, pH 7.2, and 12,000× *g*, 15 min, recovering pellet, and adding approximately 50 μL of the same buffer), using the traditional procedures with minor modifications [18,19]. The protein content of mitochondrial-enriched pellets was measured using the PierceTM BCA assay kit according to manufacturer’s instructions, using a spectrophotometer Synergy H1 (Biotek Instruments, Winooski, VT, USA).

### 2.8. Mitochondrial Complexes I and II Activities

Previously isolated mitochondria were submitted to three cycles of freezing/thawing to give substrates access to the mitochondrial matrix. Mitochondrial complex I and II activities assays were conducted at 37 °C, using up to 10 µg of mitochondrial protein, both developed in 25 mM KH2PO4 (pH = 7.5) reaction buffer, following the absorbance of DCPIP (2,6-dichloroindophenol, λ = 600 nm, ε = 20.7 × 10^−3^ M^−1^ cm^−1^), for 10 min with a 5 s read interval, using a microplate spectrofluorometer Synergy H1 (BioTek, Winooski, VT, USA). In detail, complex I (NADH-ubiquinone oxidoreductase) was evaluated spectrophotometrically, following the reduction of 0.07 mM DCPIP, according to [26] and [19], with major modifications. Buffer was previously supplemented with 1 mM KCN and 8 μM antimycin A. Immediately before starting to read the kinetics, 0.1 mM NADH was added. Thus, the time-dependent decrease of DCPIP optical density, promoted by the mitochondria-rich fraction in the absence and presence of rotenone (a specific inhibitor of complex I, at a final concentration of 0.01 mM), was recorded. Enzyme activity was determined by the difference between the slopes in the absence and presence of rotenone (to discard non-specific reduction of DCPIP), and it was expressed as nM DCPIP reduced/min/mg of protein.

Complex II (succinate-coenzyme Q reductase) activity was evaluated spectrophotometrically, following the reduction of 0.07 mM DCPIP, which is used as an exogenous final acceptor of the electrons, resulting from the succinate oxidation promoted by the enzyme [19,20]. Buffer was previously supplemented with 0.2 mM decylubiquinone, 0.01 mM rotenone, 1 mM KCN, and 8 μM Antimycin A. Immediately before starting to read the kinetics, 2 mM succinate was added. Thus, the time-dependent decrease of DCPIP optical density, promoted by the mitochondria-rich fraction in the absence and presence of oxaloacetate (compete with succinate to be catalyzed by complex II, at a final concentration of 10 mM), was recorded. Enzyme activity was determined by the difference between the slopes in the absence and presence of oxaloacetate (to discard non-specific reduction of DCPIP), and it was expressed as nM DCPIP reduced/min/mg of protein. For both activities, the calculation algorithm was based on the equation Δ[DCPIP]/min = Kinetic slope/(ε × optical path length).

### 2.9. Quantitation of Glutathione’s Levels

Reduced and oxidized glutathione levels (GSH and GSSG levels, respectively) were determined by a spectrofluorometric assay [21]. The protocol assumes the extraction of cytosolic and mitochondrial pool of glutathione. We considered 0.5 mg protein of cell pellet to one mL of a phosphate buffer (100 mM NaH_2_PO_4_, 5 mM EDTA, pH = 8) and 0.5 mL of 2.5% H_3_PO_4_. The mixture was sonicated and centrifuged at 10,000× *g*, 30 min, at 4 °C, to promote deproteinization. Supernatants were collected and neutralized with NaOH. To determine GSH content, supernatants were incubated at room temperature in 2.5 mL of phosphate buffer supplemented with 200 g orthophthalaldehyde (OPT) for 15 min. To determine GSSG content, supernatants were first incubated at 22 °C, with 12 mM N-ethylmaleimide (NEM) for 45 min. After that, 1.66 mL of 0.1 M NaOH and 0.7 mL of phosphate buffer supplemented with 200 µg OPT were added to the reaction, followed by 15 min incubation at 22 °C. Concentrations were calculated using standard curves prepared with different concentrations of GSH and GSSG, which underwent the same sample treatment. Fluorescence (Ex350/Em420 nm) was measured in a microplate spectrofluorometer Synergy H1, BioTek, Winooski, VT, USA). The results were expressed as GSH/GSSG ratio.

### 2.10. Total Protein Extraction

Protein extraction of TM4 SCs was developed overnight, using RIPA buffer previously supplemented with 1% protease inhibitor cocktail (B14002, Bimake, Houston, TX, USA), 1% of 100 mM phenylmethanesulfonyl fluoride, and 1% of 100 mM sodium orthovanadate (modified from [17]). Total protein concentration was determined using the Pierce™ BCA protein assay kit, as described previously.

### 2.11. Evaluation of Mitochondrial Complexes and Lactate Dehydrogenase Protein Levels

Quantification of protein levels of mitochondrial complexes and lactate dehydro-genase (A/C) were performed with a Western blot methodology [27] and subsequent selective immuno-detection. Total OXPHOS analyses were performed with 25 μg of total protein, mixed with sample buffer (60 mM Tris-HCl, 10% glycerol, 2% SDS, 5% β-mercaptoethanol, 0.01% bromophenol blue, pH 6.8), and denatured for 15 min at 37 °C. Proteins were fractionated in 12% polyacrylamide gels, and electrophoresis was carried out for 120 min, at 100 V. Afterwards, proteins were transferred from gels to previously activated polyvinylidene difluoride membranes (Merck Millipore, Darmstadt, Germany) in a Mini Trans-Blot^®^ cell (Bio-Rad, Hemel Hempstead, UK) and then blocked for 3 h in a 5% non-fat milk solution at room temperature. The membranes were incubated overnight at 4 °C, with a mouse anti-rodent total OXPHOS antibody cocktail (1:1000, ab110413, Abcam, UK). The antibody mixture was the purpose for targeting five mitochondrial proteins epitopes: NDUFB8 (NADH dehydrogenase (ubiquinone) 1 beta subcomplex subunit 8), representing complex I; SDH8 (succinate dehydrogenase assembly factor 4), complex II; UQCRC2 (cytochrome b-c1complex subunit 2), complex III; MTCO-1 (mitochondria-encoded cytochrome c oxidase I), complex IV; and ATP5A (ATP synthase F1 subunit alpha), complex V. As a protein-loading control and band normalization, Ponceau S (5%) staining was used. The immune-reactive proteins were detected with goat anti-mouse antibody (1:5000, A3562, Sigma-Aldrich, Taufkirchen, Germany). Membranes were reacted with WesternBright™ ECL and visualized with the Bio-Rad ChemiDoc XR (Bio-Rad, Hemel Hempstead, UK). Densities from each band were calculated using the Image Lab Software (Bio-Rad, Hemel Hempstead, UK), normalized to Ponceau S total protein band) [17]. LDH detection comprised the same protocol, but primary antibody overnight incubation was performed with rabbit anti-mouse LDH (1:15000, an-ti-LDHA/LDHC, C28H7, Cell Signaling Technology, USA), and immune reactive proteins were detected with goat anti-rabbit IgG (1:5000, A3687, Sigma-Aldrich, Taufkirchen, Germany).

### 2.12. Reverse Transcriptase Polymerase Chain Reaction (RT-PCR)

Extraction of total RNA (tRNA) from TM4 cells was performed using the NZY total RNA isolation kit (NZYTech, Oeiras, Portugal), as indicated by the manufacturer protocol [17]. tRNA obtained for each sample was reversely transcribed using the NZY M-MLV RT. The resulting complementary DNA (cDNA) was used with exon–exon spanning primer sets designed [28] to amplify Ar (androgen receptor), B2m (Beta-2-microglobulin), Il-6v1 (Interleukin 6, transcript variant 1), and Nr3c1 (glucocorticoid receptor) transcripts (Table 1). Each polymerase chain reaction (PCR) contained 1 μL of cDNA in 12.5 μL of final volume of a mixture containing 6.5 μL of NZYTaq green master mix, 0.1 µL (50 μM) of each primer, and sterile H_2_O up to 20 μL. Mouse testis tissue was used as positive control, and cDNA-free sample was used as a negative control. At the end of the experiments, samples were run in 2% agarose gel electrophoresis with 2 μL of Greensafe in 200 mL, for 30 min at 120 V. Gels were visualized and analyzed in a Bio-Rad GelDoc XR (Bio-Rad, Hemel Hempstead, UK) using the Quantity One software (Bio-Rad, Hemel Hempstead, UK). The sizes of the expected products were compared to a DNA ladder.

### 2.13. Evaluation of mRNA Transcripts Levels of Glucocorticoid Receptor, Androgen Receptor and Interleukin-6 by Quantitative PCR (qPCR)

mRNA expression levels of Ar, Il-6v1, and Nr3c1 were evaluated by qPCR [17]. The specific primers (Table 1) were again used to semi-quantify the transcripts. qPCR was carried out in a CFX Connect™ real-time PCR system (Bio-Rad, Hercules, CA, USA), using unskirted low-profile 96-well PCR plates (Bio-Rad, Hercules, CA, USA) and considering SYBR^®^ green as fluorescent dye. Amplification efficiency was determined for all primer sets using serial dilutions of cDNA. qPCR amplifications of cDNA targets considered per reaction 1 µL of diluted 1:15 cDNA,10 μL NZY qPCR green master mix, 0.8 μL (10 mM) of forward and reverse primers for each gene, and sterile H_2_O up to 20 μL final. Amplification conditions comprised an initial denaturation step of 5 min at 95 °C, followed by 40 (*Ar*, *Il-6* and *Nr3c1*) or 35 (*B2m*) runs of a 3-step cycle: denaturation—30 s, at 95 °C; annealing—30 s, at a specific temperature (see Table 1); and extension—1 min, at 72 °C. B2m transcript levels were used as a reference gene to normalize the mRNA expression of target genes. Fold variation of the expression levels was calculated following the mathematical model proposed by Pfaffl (considering the formula: 2^−ΔΔCt^ [29]) and normalized to data from untreated cells.

### 2.14. Statistical Analysis

Experimental results comprised at least three or more biological replicates (additional technical replicates were considered, when mentioned). Corticosterone-free condition (CORT-free) was considered, when mentioned, to normalize data in independent biological replicates. Data were graphically presented in most as box plots (with median, minimum, and maximal values, as well as total points and first and third quartiles) or as mean ± SEM. Statistical analysis was performed using GraphPad Prism 8.0.1 (GraphPad Software, San Diego, CA, USA), considering by routine an ordinary One-way ANOVA, with a Tukey’s multiple comparison test. Data with *p* < 0.05 were considered statistically different.

## 3. Results

We evaluated the impact of different corticosterone concentrations in TM4 SCs metabolic performance and physiology. Prior to the protocols described above, viability assays (data not shown) were performed, showing that none of the concentrations of corticosterone impacted SCs survival or proliferation.

### 3.1. Corticosterone Did Not Cause an Alteration on Androgen Receptor and Glucocorticoids Receptor Transcript Levels in Sertoli Cells

**Figure 1:**
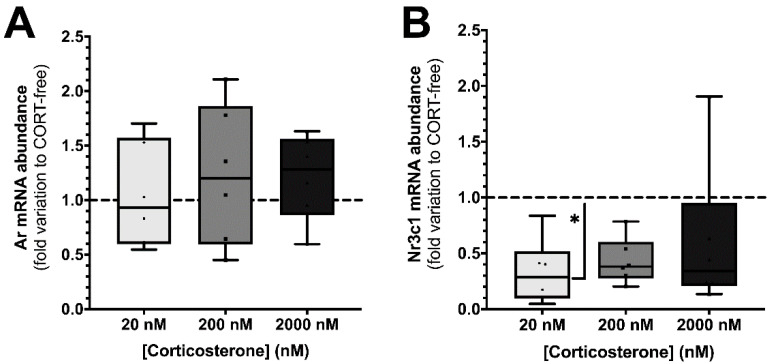
Effect of corticosterone (20, 200, and 2000 nM) treatment in mouse SCs (TM4) *Ar* (panel (**A**)) and *Nr3c1* (panel (**B**)) transcript levels. Corticosterone-free condition (CORT-free) was taken into account to normalize data. Ordinary one-way ANOVA, with a Tukey’s multiple comparison test, was used for statistical analysis. Results are expressed as box plots (with median, minimum, and maximal values, as well as total points and first and third quartiles) (n = 6 for each condition). Data with *p* < 0.05 were considered statistically different (* *p* < 0.05).

The effects of corticosterone on gene expression of relevant receptors, AR and NR3C1/GR (glucocorticoid receptors), were addressed by qPCR, as presented in Figure 1. The exposure to the three concentrations of the hormone did not significantly alter the transcription of *Ar* and *Nr3c1* genes in TM4 SCs (Figure 1); interestingly, the *Nr3c1* transcripts levels were all slightly depressed (median under 1, regarding fold variation), and the *Ar* transcripts were mildly increased (median equal or above 1), as compared to the levels observed in absence of corticosterone.

When TM4 SCs were exposed to 20, 200, and 2000 nM corticosterone the mRNA levels of *Ar* gene were 1.04 ± 0.20, 1.23 ± 0.26, and 1.21 ± 0.16-fold variation to CORT-free conditions (Figure 1, panel A), respectively, while those of *Nr3c1* transcripts decreased to 0.32 ± 0.12, 0.43 ± 0.08, and 0.60 ± 0.27-fold variation to CORT-free conditions (Figure 1, panel B), respectively.

### 3.2. Corticosterone Impacted the Metabolic Profiling of Sertoli Cells

**Figure 2:**
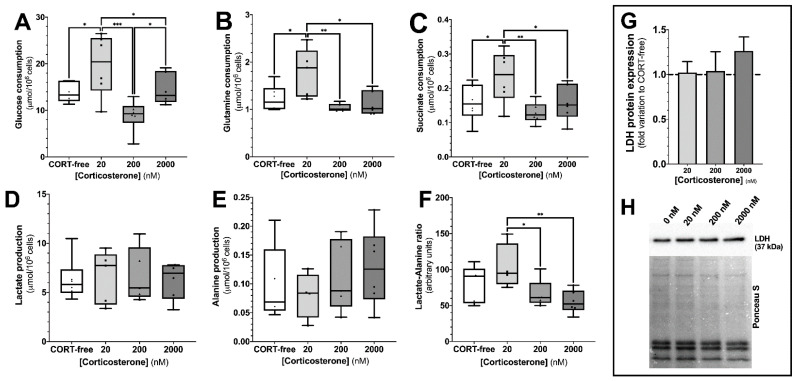
Effect of corticosterone (20, 200, and 2000 nM) treatment in mouse SCs (TM4) consumption and production of the 1H-NMR detectable metabolites glucose, glutamine, lactate, succinate, and alanine (Panels (**A**–**E**)), as well as lactate/alanine ratio (Panel (**F**)) and LDH protein expression (semi-quantitation–Panel (**G**); representative membrane–Panel (**H**)). Corticosterone-free condition (CORT-free) was considered to normalize data (Panel (**G**)). Ordinary one-way ANOVA, with a Tukey’s multiple comparison test, was used for statistical analysis. Results are expressed as box plots (with median, minimum, and maximal values, as well as total points and first and third quartiles)—panels (**A**–**F**) (n = 6 for each condition) and mean ± SEM-Panel (**G**) (n = 5 for each condition). Data with *p* < 0.05 were considered statistically different (* *p* < 0.05; ** *p* < 0.01; *** *p* < 0.001).

By following the extracellular metabolic secretome, it was possible to observe the profile of glucose, glutamine, and succinate consumption (Figure 2, panels A–C). SCs exposed to the sub-physiological concentration of corticosterone (20 nM) presented a strong increase in the consumption of these three metabolites, as compared to the cells from all the other groups. When the cells were exposed to 20 nM of corticosterone for 24 h, they consumed 19.7 ± 2.7 μmol of glucose/million of cells (Figure 2, panel A), which was significantly higher than the consumption by the cells under CORT-free conditions (13.8 ± 0.9 μmol of glucose/million of cells). The consumption of glucose was also significantly higher in the SCs exposed to the supra-physiological concentration of corticosterone (2000 nM) (14.5 ± 1.4 μmol of glucose/million of cells) than that of cells exposed to the physiological concentration of corticosterone (200 nM) (8.9 ± 1.4 μmol of glucose/million of cells).

An analogous trend was observed in the consumption of glutamine and succinate. SCs exposed to 20 nM of corticosterone consumed significantly more glutamine (1.78 ± 0.23 μmol/million of cells) than the cells exposed to CORT-free conditions (1.23 ± 0.11 μmol/million of cells), as well as the physiological and supra-physiological concentrations of corticosterone (1.03 ± 0.03 and 1.12 ± 0.10 μmol/million of cells, respectively) (Figure 2, panel B). Similarly, the cells exposed to the sub-physiological concentration of corticosterone (20 nM) consumed significantly more succinate (0.23 ± 0.03 μmol of glucose/million of cells) than the cells exposed to CORT-free conditions (0.15 ± 0.02 μmol/million of cells), with 200 nM corticosterone (0.13 ± 0.01 μmol/million of cells) and 2000 nM corticosterone (0.16 ± 0.02 μmol/million of cells) (Figure 2, panel C).

In contrast, the lactate and alanine production by SCs (Figure 2, panels D,E) was not significantly altered in the presence of corticosterone; however, the lactate/alanine ratio presented an inverse trend to that of corticosterone concentration (Figure 2, panel F). Cells from the groups exposed to CORT-free conditions (6.31 ± 0.08 μmol of lactate/million of cells; 0.10 ± 0.03 μmol of alanine/million of cells), and 20 nM corticosterone (6.60 ± 1.20 μmol of lactate/million of cells; 0.10 ± 0.03 μmol of alanine/million of cells), 200 nM corticosterone (6.75 ± 1.25 μmol of lactate/million of cells; 0.11 ± 0.03 μmol of alanine/million of cells), and 2000 nM corticosterone (6.24 ± 0.77 μmol of lactate/million of cells; 0.13 ± 0.03 μmol of alanine/million of cells) presented similar production to lactate and alanine (Figure 2, panels D,E). As concerning lactate/alanine ratio, there was a decrease in the cells treated with the physiological (66 ± 9 arbitrary units) and supra-physiological (55 ± 7 arbitrary units) concentrations, as compared to the ones exposed to 20 nM corticosterone (104 ± 16 arbitrary units), which was not statistically different from the ratio observed in the cells from the CORT-free group (80 ± 12 arbitrary units) (Figure 2, panel F). As lactate is a key metabolite in spermatogenesis, we also evaluated the impact of corticosterone on lactate dehydrogenase protein levels, being that the quantity of this enzyme was not affected by exposure of the SCs to any of the concentrations used (Figure 2, panels G,H).

### 3.3. Corticosterone Modulated Mitochondrial Complex II Activity in TM4 Sertoli Cells

**Figure 3:**
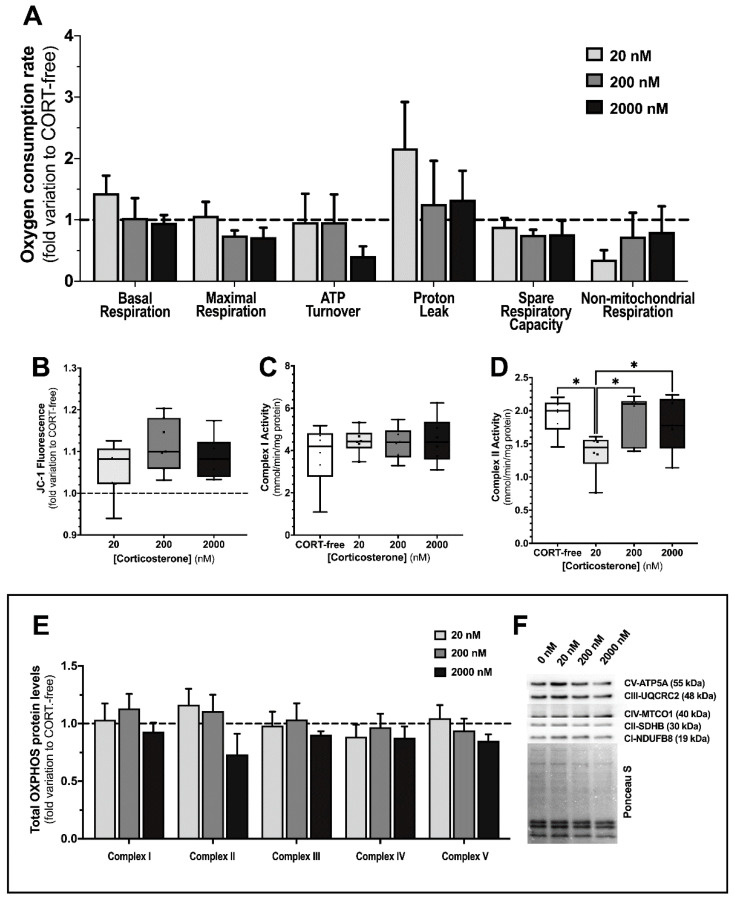
Effect of corticosterone (20, 200, and 2000 nM) treatment in mouse SCs (TM4) intact cell oxygen consumption rates (Panel (**A**)) and mitochondrial membrane potential (ΔΨ relative values by mean of JC-1 fluorescence aggregates/monomers ratio (Panel (**B**)). Mitochondrial complexes I and II enzymatic activity (Panels (**C**,**D**)) and total OXPHOS protein expression (semi-quantitation–Panel (**E**); representative membrane–Panel (**F**)). Corticosterone-free condition (CORT-free) was taken into account to normalize data in Panels (**A**,**B**,**E**). Ordinary one-way ANOVA, with a Tukey’s multiple comparison test, was used for statistical analysis. Results are expressed as mean ± SEM—Panels (**A**) (n = 4–9, different respiratory parameters) and (**E**) (n = 5 for each condition)—or box plots (with median, minimum, and maximal values, as well as total points and first and third quartiles)—Panels (**B**) (n = 3 for each condition, with 6 technical replicates), (**C**,**D**) (n = 4 for each condition). NDUFB8 (NADH dehydrogenase (ubiquinone) 1 beta subcomplex subunit 8), complex I; SDH8 (succinate dehydrogenase assembly factor 4), complex II; UQCRC2 (cytochrome b-c1complex subunit 2), complex III; MTCO-1 (mitochondria-encoded cytochrome c oxidase I), complex IV; and ATP5A (ATP synthase F1 subunit alpha), complex V. Data with * *p* < 0.05 were considered statistically different.

The full assessment of the mitochondrial electron chain, in an integrated view, was established by means of oxygen consumption recording (Figure 3, panel A). In general, no alteration was perceived in the vast majority of the mitochondrial functional parameters assessed for the cells exposed to corticosterone, as compared with SCs under CORT-free condition. Still, when the cells were exposed to the sub-physiological dose of corticosterone, we observed a tendency to enhance the majority of the respiratory parameters, as evidenced in the *basal respiration* in line, with *non-mitochondrial respiration*. Overviewing the SCs exposed 2000 nM corticosterone, and data show a slight negative impact on *ATP turnover* (defined as the ratio between ATP content and ATP production, and the capacity of ATP synthase to maintain the phosphorylation of ADP); however, regarding mitochondrial membrane potentials (ΔΨ), no differences were observed (Figure 3, panel B) in SCs exposed to any of the assayed corticosterone concentrations. In general, oxidative phosphorylation was not impacted as a whole, but small changes where observed. In fact, the screening of protein levels of all mitochondrial complexes (Figure 3, panels E,F) showed no alterations when cells were exposed to corticosterone. Still, when assessing mitochondrial complexes activities, slight alterations were perceived in mitochondrial complex I activity of SCs exposed to corticosterone (Figure 3, panel C); when TM4 SCs were exposed to 20 nM corticosterone, a lower complex II activity was seen (1.36 ± 0.13 mmol/min/mg protein), as compared with that observed in cells exposed to CORT-free conditions (1.92 ± 0.11 mmol/min/mg protein), 200 nM corticosterone (1.81 ± 0.15 mmol/min/mg protein), and 2000 nM corticosterone (1.80 ± 0.19 mmol/min/mg protein) (Figure 3, panel D).

### 3.4. Corticosterone Had No Impact on Redox Status of Sertoli Cells

**Figure 4:**
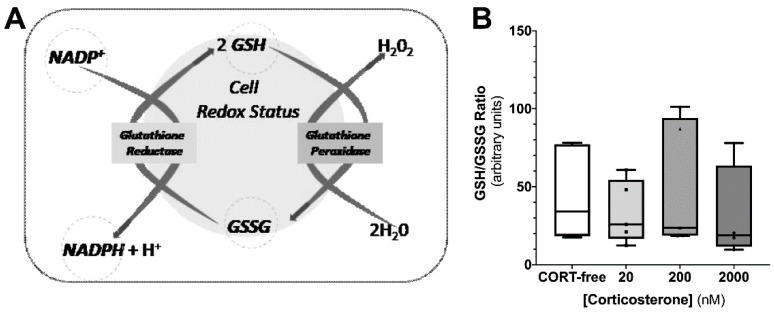
Effect of corticosterone (20, 200, and 2000 nM) treatment in mouse SCs (TM4) on reduced glutathione/oxidized glutathione ratio, as an indirect measurement of cell redox power (as outlined in the panel (**A**)). Corticosterone-free condition (CORT-free) was considered to normalize data (panel (**B**)). Ordinary one-way ANOVA, with a Tukey’s multiple comparison test, was used for statistical analysis. Results are expressed as box plots (with median, minimum, and maximal values, as well as total points and first and third quartiles)—(n = 3 for each condition, with 3–9 technical replicates). Data with *p* < 0.05 were considered statistically different.

It was possible to indirectly address the redox status (power) of TM4 SCs exposed to corticosterone, considering a simple interplay between the NADPH/NADP^+^ and GSH/GSSG ratios (Figure 4, panel A), orchestrated mainly by the enzymes glutathione reductase and glutathione peroxidase. Increasing concentrations of corticosterone slightly impacted glutathione pool in these cells, by means of the reduced/oxidized forms ratio, but not enough to consider that corticosterone increased oxidative stress and decrease in general redox power. In fact, cells exposed to CORT-free conditions (45 ± 13 arbitrary units), i.e., 20 nM corticosterone (36 ± 9 arbitrary units), 200 nM corticosterone (50 ± 18 arbitrary units), and 2000 nM corticosterone (31 ± no16 arbitrary units), presented similar reduced/oxidized glutathione ratios (Figure 4, panel B).

### 3.5. Corticosterone Had a Strong Impact on Interleukin-6 Transcript Levels in Sertoli Cells

Concerning *Il6*, exposure to increasing concentrations of corticosterone caused an increasing impact on the mRNA levels of this cytokine. Indeed, we observed an inverse relationship between the corticosterone concentration and *Il6* mRNA levels.

**Figure 5:**
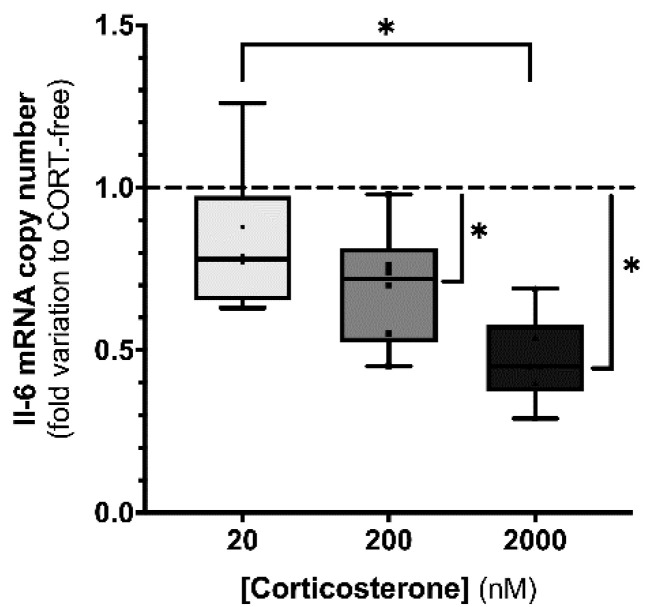
Effect of corticosterone (20, 200, and 2000 nM) treatment in mouse SCs (TM4) *Il6* transcript levels. Corticosterone-free condition (CORT-free) was taken into account to normalize data. Ordinary one-way ANOVA, with Tukey’s multiple box plots, was used for statistical analysis. Results are expressed as box plots (with median, minimum, and maximal values, as well as total points and first and third quartiles) (n = 6 for each condition). Data with * *p* < 0.05 were considered statistically different.

The decrease in the transcription of the gene allocated for the interleukin-6 transcript levels was the highest in SCs exposed to 2000 nM corticosterone (0.47 ± 0.06-fold variation to CORT-free conditions), with them also being perceivable in the cells exposed to 200 nM (0.70 ± 0.07-fold variation to CORT-free conditions) and 20 nM corticosterone (0.83 ± 0.09-fold variation to CORT-free conditions) (Figure 5).

## 4. Discussion

Stress is a phenomenon that occurs throughout the human lifespan, which, in punctual situations can be positive, but if it is chronic, it can affect the individual’s wellbeing. Events that trigger stress are called stressors, and these can be of either external or internal origin, with glucocorticoid hormones playing a pivotal role in the response to multiple stressors [30]. Corticosterone is the main corticosteroid hormone in rats and mice, being reported throughout the literature as having a multimodal action, with its concentration being a crucial parameter to differentiate its role for specific tissues [31]. Indeed, it has been suggested that corticosteroids negatively impact male fertility, with a particular focus on Sertoli cells (SCs) [32,33], although it is also known that, for a regular male reproductive function, residual levels of glucocorticoids and the activation of its specific receptors are required [1]. In this work we aimed to evaluate the impact of increasing concentrations of corticosterone on the physiology of SCs, particularly on its glycolytic and mitochondrial performance, as well as its autoimmune activity, through the expression of IL-6.

In our work, we used SCs monocultures, a simpler, but effective, approach to evaluate autocrine and paracrine activity at the cell levels [34] that were exposed to sub- (20 nM), iso- (200 nM), and supra-physiological (2000 nM) concentrations of corticosterone [13,14,15] in a FBS-free medium to guarantee the existence of a corticosterone-free condition (CORT-free). We firstly assessed the transcription levels of androgen (AR) and glucocorticoid (GR) receptors in TM4 SCs exposed to different corticosterone concentrations, in which, no alteration on the expression levels of AR was seen. Indeed, it seems that the 24-h treatment of TM4 SCs with the selected concentrations of corticosterone was not able to revert the phenotype of these cells, concerning the response mechanisms to androgens, a key aspect of SCs physiology. Still, the expression levels of GR were responsive to the exposure to corticosterone, with lower levels of mRNA transcripts in the cells exposed to the highest dose of corticosterone. It has been reported that glucocorticoids are able to autoregulate the mRNA and/or protein levels of its receptor by distinct feedback mechanisms, either by directly impacting the mRNA levels or by an indirect mechanism that involves degradation of the protein in the proteasome [35,36]. In our TM4 SCs, direct feedback seems to be present, as previously described in lymphoblastoid cell lines [36]. It has been suggested that this autoregulation mechanism may be a central aspect in the physiological response to stress, particularly in the modulation of glucocorticoid-dependent catabolic and anabolic processes [35,36]. Hence, given the extreme relevance of SCs metabolism for the development of spermatogenesis [37] and its sensitivity to various endogenous and exogenous factors [38,39], we analyzed the impact of this glucocorticoid on the extracellular metabolic fluxes of SCs, allowing us to infer, even if only partially, the relationship between glycolysis, the Krebs cycle, and the level of mitochondrial activation when these cells were challenged with corticosterone.

It has been reported that glucocorticoids elicit a myriad of distinct metabolic outcomes, depending on the organ or tissue it acts on. For instance, in the skeletal muscle, glucocorticoids decrease glucose uptake (antagonizing insulin) and metabolization, whereas, in the liver, they promote gluconeogenesis [40]. In the testis, it has been described that corticosterone promotes a decrease in lactate content, which might be due to its lower production by SCs or higher utilization by developing germ cells [32]. In our work, when TM4 SCs are exposed to the chosen concentrations of corticosterone for 24 h, we observed that the consumption of glucose, glutamine, and succinate were increased only in the cells exposed to the sub-physiological concentration of this glucocorticoid (20 nM), while remaining constant in the cells from the other groups. Still, in the cells exposed to 20 nM corticosterone (as in the cells from the other groups), no alteration in lactate production was seen (as well as no alteration in the LDH protein levels), which led us to suggest that TM4 cells might be stimulated to increase the performance of the Krebs cycle (in a catabolic perspective) to feed the mitochondrial respiratory chain. The lactate/alanine ratio helped us to further highlight this hypothesis, considering that this ratio is associated with the cellular redox state, by means that pyruvate, is converted to lactate (or alanine) and coupled with the concomitant oxidation/reduction of NADH to NAD+ [41,42]. The observed increase in the lactate/alanine ratio observed in the SCs exposed to the sub-physiological concentration of corticosterone indicates a rise in the reducing potential, which would ultimately feed the mitochondrial respiratory chain.

When screening the full action of the mitochondrial respiratory chain and mitochondrial membrane potential, by means of oxygen consumption and mitochondrial potential (to address oxidative phosphorylation, OXPHOS), the effects observed were quite tenuous for all corticosterone concentration assayed. Indeed, few studies were considered to access the corticosterone effects on the mitochondria. An in vivo study with a reptile [43], using metabolic cages, as well as isolated liver mitochondria, reported no correlation of this hormone augmentation with some respiratory parameters; it additionally observed positive alterations on ATP content, although the ATP pool in the cells is not a consensual parameter. Regarding that, in a “fight or flight” response mode, a huge energy demand to tissues, such as the liver and muscle, occurs; however, in peripheral tissues, such as the testis, no studies reported the respiratory behavior as a whole. Our results reported that TM4 maintained the background functioning of mitochondrial activity, where the coupling of ATP synthesis, by means of the mitochondrial membrane potential and respiratory parameter ATP turnover, had been slightly pinched in the highest concentration of corticosterone assayed, as well as in the tendency to increase non-mitochondrial respiration phenomena. While the expression of mitochondrial complexes proteins was not altered in any of the conditions assayed, there was a decrease on mitochondrial complex II activity in the SCs exposed to the sub-physiological concentration of corticosterone. Still, this effect seems to have been dimmed by the cells, for they were able to maintain the background functioning of mitochondrial activity. This suggests that in regular conditions SCs mitochondria might not be working in full, thus safeguarding the existence of detrimental stimuli and need to enhance mitochondrial activity to sustain the spermatogenic potential. Moreover, in cells, the glutathione redox couple GSH/GSSG has a major role and together with other redox-active couples, including NADPH/NADP+, which regulates and maintains the appropriate cellular redox status [44]. Changes in the GSH/GSSG ratio are fundamental in the fine-tuning of redox power transduction. For instance, transient increases of reactive oxygen species (ROS) have been described to induce an increase on GSH levels aiming at redox status restauration; however, when oxidative stress becomes prolonged and cell resources are no longer able to counteract the oxidative-mediated challenges, the GSH pool substantially decrease, impacting several cellular processes, thus leading to cell malfunction and, later on, a deathly fate [44]. Additionally, the relationship between the GSH/GSSG and NADPH/NADP+ ratios in cytosol or mitochondrial matrix are both indirectly connected by membrane shuttles that lately maintain the “fluxes” of redox players. Our results disclose a maintenance of the GSG/GSSG ratio in response to the assayed corticosterone concentration, which is in accordance with the oxygen consumption parameters observed in SCs.

Lastly, we evaluated Il6 expression levels, considering that IL-6 is a recognized signaling cytokine in inflammation processes triggered by glucocorticoids. SCs are known to produce IL-6 in a FSH and stage dependent manner [10,45]. Several roles have been attributed to IL-6 within the testis, suggesting that this cytokine is an important autocrine and paracrine regulator [46]. It has been reported that, in the testis, IL-6 inhibits DNA synthesis during the seminiferous epithelium cycle, modulates the secretion of both inhibin B and transferrin by SCs, and is able to reduce the motility of spermatozoa [11]. The observed decrease in Il6 transcript levels is in agreement with a previously published work in rat testicular tissue [47]. Still, the deviation from “non-stress” corticosterone, in terms of Il6 transcription, was a false question in the previous model. Monocultures outputs, where cells are uprooted from their original tissue environment, may not reflect the organ behavior when serum corticosterone doses were deviated from physiological levels, as described for in vivo studies. Additionally, the treatment time (24 h) and absence of a circulatory system restrain the direct translation of the results to the real perception of corticosterone effects on animal/human testicular tissues [9]. In fact, for in vitro studies using hormone-responsive cells, such as SCs, addressing the proper corticosterone concentration is of maximal importance. In fact, a previous study evaluated the impact of the distinct doses of this glucocorticoid using FBS-containing cultured medium [32], which did not guarantee a CORT-free condition at the start and hampers correlations with our study. FBS is known to provide a cocktail of multiple components, including several glucocorticoids [48].

It is important to highlight two types of actions: the ones regarding relatively fast mechanisms (such as interleukin-6 expression and the potential release of the surroundings) and the metabolic features associated with energy production. In this last one, ATP demand in the stress response is divided into a known fight or flight response (acute action) or sustained marathon to reach a metabolic steady state (chronic condition). Our experimental paradigm may be considered to be in an intermedium state, considering the mature cells in a confined static medium, and the metabolite consumption/secretion highlight a declared hormesis effect around the value, assumed as physiological, thus stressing that the TM4 cells fine-tuned the responses to specific hormonal challenges, increased substrates consumption to upturn fast ATP synthesis, and prepared cells to a rescue mode.

## 5. Conclusions

Our work provides new insights regarding the regulation of SCs metabolism under the influence of glucocorticoids by dissecting cell multitargets of corticosterone action/repercussion, avoiding the interference of the hypothalamic-pituitary-gonadal axis, and providing clues to scale up to more complex biological structures. The hormesis effect observed in some bioenergetic parameters is the major take-home message, when considering the monoculture and treatment timeframe as the experimental biological paradigm. Nevertheless, further knowledge on the functioning and regulation of these biochemical mechanisms is essential for the enlightenment of a process that is central to spermatogenesis and fertility. The addressment of other metabolic parameters, as well as the intermediary players, though outside the scope of this work, may help us to further understand the role of glucocorticoids on male fertility. Athletes, or even men whose jobs include high levels of responsibility or danger, can have chronic high levels of cortisol (human homologues of corticosterone) and be important subjects for future studies.

## Figures and Tables

**Table 1:** Accession number, oligonucleotide sequence, and respective conditions for PCR amplification of targeted mRNAs from mouse Sertoli cells (TM4 cells).

Target (*Accession Number*)	Sequence (5′-3′)	AT (°C)	Number of Cycles	Specie of Origin
***Ar*** (NM_013476.4)	*FWD: GCTCACCAAGCTCCTGGATT*	60	40	*Mus musculus*
	*RVS: TCAGGAAAGTCCACGCTCAC*			
***B2m*** (NM_009735.3)	*FWD: ACGTAACACAGTTCCACCCG*	58	35	*Mus musculus*
	*RVS: TCTCGATCCCAGTAGACGGT*			
***Il-6v1*** (NM_031168.2)	*FWD: TGAGAAAAGAGTTGTGCAATGG*	60	40	*Mus musculus*
	*RVS: GGAGAGCATTGGAAATTGGGG*			
***Nr3c1*** (NM_008173.4)	*FWD: GTGGAAGGACAGCACAATTACC*	60	40	*Mus musculus*
	*RVS: GAGACTCCTGCAGTGGCTTG*			

Abbreviations: *Ar* (androgen receptor); AT (annealing temperature); *B2m* (beta-2-microglobulin); FWD (forward); *Il-6v1* (interleukin 6, transcript variant 1); *Nr3c1* (glucocorticoid receptor); RVS (reverse).

## Data Availability

Not applicable.
